# A scientometric analysis of neuroblastoma research

**DOI:** 10.1186/s12885-020-06974-3

**Published:** 2020-05-29

**Authors:** Illya Martynov, Jessica Klima-Frysch, Joachim Schoenberger

**Affiliations:** 1 Department of Pediatric Surgery, University Hospital of Freiburg, Freiburg, Germany; 2 Department of Pediatric Surgery, University of Leipzig, Leipzig, Germany

**Keywords:** Neuroblastoma, Scientometrics, Research performance, Children, Network analysis

## Abstract

**Background:**

Thousands of research articles on neuroblastoma have been published over the past few decades; however, the heterogeneity and variable quality of scholarly data may challenge scientists or clinicians to survey all of the available information. Hence, holistic measurement and analyzation of neuroblastoma-related literature with the help of sophisticated mathematical tools could provide deep insights into global research performance and the collaborative architectonical structure within the neuroblastoma scientific community. In this scientometric study, we aim to determine the extent of the scientific output related to neuroblastoma research between 1980 and 2018.

**Methods:**

We applied novel scientometric tools, including Bibliometrix R package, biblioshiny, VOSviewer, and CiteSpace IV for comprehensive science mapping analysis of extensive bibliographic metadata, which was retrieved from the Web of ScienceTM Core Collection database.

**Results:**

We demonstrate the enormous proliferation of neuroblastoma research during last the 38 years, including 12,435 documents published in 1828 academic journals by 36,908 authors from 86 different countries. These documents received a total of 316,017 citations with an average citation per document of 28.35 ± 7.7. We determine the proportion of highly cited and never cited papers, “occasional” and prolific authors and journals. Further, we show 12 (13.9%) of 86 countries were responsible for 80.4% of neuroblastoma-related research output.

**Conclusions:**

These findings are crucial for researchers, clinicians, journal editors, and others working in neuroblastoma research to understand the strengths and potential gaps in the current literature and to plan future investments in data collection and science policy. This first scientometric study of global neuroblastoma research performance provides valuable insight into the scientific landscape, co-authorship network architecture, international collaboration, and interaction within the neuroblastoma community.

## Background

Neuroblastoma (NB) is the most common extracranial malignant pediatric tumor that typically arises in the adrenal medulla or paraspinal sympathetic ganglia [1]. The histological differentiation state of NB is highly variable, including undifferentiated “small blue round cell” neoplasms, partial differentiated ganglioneuroblastomas (GNB), and differentiated ganglioneuroma (GN), which consists of clusters of mature neurons surrounded by a dense stroma of Schwann cells. As an immature tumor, NB is aggressive, predominantly occurring in early childhood at a median age of 22 months and accounting for 15% of childhood cancer-related mortality. The overall survival rate for high-risk metastatic disease is 40% [2–5]. Conversely, mature variants (GNB or GN) occur in older children and tend to behave in a more benign fashion [6].

In addition to tumor histology, many molecular genetic markers of NB have been identified, including amplification of the N-myc proto-oncogene protein (MYCN), mutations of the anaplastic lymphoma kinase (ALK) receptor, allelic deletions in the 1p, 3p and 11q chromosomal regions, chromosomal gain of 17 or tumor cell ploidy [7, 8]. Amplification of the MYCN gene is associated with poor prognosis and was found in about 20% of NB cases [9, 10]. ALK is altered by gain-of-function point mutations in around 14% of high-risk NB and confers poorer prognosis for tumors in the intermediate- and high-risk categories [11, 12].

Treatment regimens for patients with NB differ accordingly and depend on tumor behavior as predicted by tumor histology and molecular features [13]. Children with low-risk NB can be observed or treated surgically while those with intermediate risk disease may receive chemotherapy prior to surgical resection. Patients with high-risk NB undergo intensive multimodal therapy including chemotherapy, surgical treatment, stem cell transplantation, radiation, and immunotherapy [14, 15].

Over the past decades, national and international collaborative research efforts have led to increased knowledge of biological and clinical tumor features, thereby refining patient’s risk stratification and treatment strategies, leading to significant increases in survival rates. Currently, patients with low- and intermediate-risk NB have an overall survival rate of about 90% [16, 17]. However, children with high-risk NB still have a poor prognosis [3]. Even if NB was successfully treated, disease burden persists, as the NB survivors have long-term health consequences due to damage of the organ systems by chemotherapy and radiation therapy. Nearly two thirds of NB survivors have at least one chronic health condition and one third have severe to life-threatening illness [18, 19]. To improve understanding of the genetic basis of NB, the neuroblastoma research community has collected large numbers of tumor and germline samples. With this, key somatic and germline genomic alterations have been discovered. These collective advancements have led to the development of new therapeutic approaches for high-risk NB [20].

Given the enormous volume, heterogeneity and variable quality of NB-related publications, an assessment of the scientific literature on this topic is essential for both clinicians and researchers. Hence, we employed scientometric methodologies and innovative visualization tools to analyze extensive bibliographic metadata related to NB research.

The study objectives are: 1) to assess the publication output as proxy for productivity of a researcher (quantity indicator); 2) to gauge the impact of the research on the scientific community by analysis of citation dynamics in NB research during 1980–2018 (quality indicator); 3) to identify and characterize the most prolific authors; 4) to examine the academic journals publishing papers related to NB; 5) to examine geographical distribution of the research performance on NB; 6) to analyze the co-authorship network architecture; 7) to identify the most cited NB papers; 8) to perform a keyword analysis.

## Methods

All peer-reviewed scientific publications relating to NB research were retrieved from the Web of Science™ Core Collection Database (Clarivate Analysis, Boston, USA). The search terms {“neuroblastoma(s)”} OR {“ganglioneuroblastoma(s)”} OR {“ganglioneuroma(s)”} OR {“peripheral neuroblastic tumor(s)”} were used in the title field and results were filtered by publication year from 1980 through 2018. No language restrictions were imposed. The complete metadata for each original publication and review article was compiled and manually exported on November 12, 2019. The “citation report” function from Web of Science was applied to assess citation rates and *h*-index.

Bibliometrix (version 1.7), an R-Tool of R-Studio (Version 3.6.1) for comprehensive science mapping analysis, and biblioshiny, the shiny interface providing a web-interface for bibliometrix, were used to import and manage the metadata from Web of Science™ [21]. Baseline metadata included print features, such as author’s name, corresponding author’s country (CAC), total number of publications, citations count with total citations (TC), average article citations (AAC), number of citing articles with and without self-citations, journal sources, keywords, countries/regions, and the author-level metrics such as *h*-, *m*-, and *g* indices. The *h*-index, a common proxy measure for individual scientific output, is defined as the number of papers with citation number ≥ *h* (at least one citation) [22]. Consequently, the *h*-index depends on both the number of a scientist’s publications and their impact on peers (number of citations). Since the *h* -index does not account for the career span of the author, the *m*-index or *m*-quotient (equal to the *h*-index divided by the number of years since the author’s first publication [m-quotient = *h*-index/n, *n* = number of years since the first published paper of the scientist]) was applied. Further, to account for the citation evolution of the most cited papers of the given author over time, the *g*-index, which gives credit for the most highly cited papers in a data set, was used. The annual growth rate of scientific publications was assessed applying a calculator available at www.investopedia.com/calculator/cagr.aspx.

Collaboration measures included the number of documents per author (documents/author), number of authors per document (authors/document), and number of co-authors per document (author’s appearance/documents).

In addition, using the word co-occurrence in our bibliographic data collection, we mapped the conceptual structure of an entire word’s framework with a dimensionality reduction technique and Multiple Correspondence Analysis (MCA) [23] We identified clusters of documents which express common concepts. Words appearing together in an article were related in a network.

VOSviewer (version 1.6.13, http://www.vosviewer.com), a network analysis software tool, was used to construct a keyword co-occurrence network [24]. The co-occurrence of two keywords reflects the number of publications in which both keywords occur together. The size of the circles in the VOSviewer diagram indicates the number of publications that have the corresponding keywords. The link strength between the circles reflects the frequency of keyword’s co-occurrence. The total link strength is the sum of link strengths of the keyword over all the other keywords.

CiteSpace IV (Drexel University, Philadelphia, PA, USA, Version 0.65) was applied to determine the keywords with strong citation bursts, which serves as an indicator of the most active area of research attracting a special degree of attention from the scientific community. Relationships between author’s keywords, references used, and the top authors were summarized by a Sankey plot (three-fields plot).

Categorical variables were expressed as frequency and percentage, continuous variables were represented as medians with maximum and minimum or as means with standard deviation. The Spearman correlation coefficient was used to test correlations between selected continuous variables. Statistical analyses were performed with SPSS v. 23 (SPSS 23.0 – SPSS Inc., Chicago Illinosis) and GraphPad Prism v. 6.01 (GraphPad, La Jolla, CA). All tests were two-sided. *P*-values of < 0.05 were considered statistically significant. This study did not require approval of an ethics committee.

## Results

### Overall publication performance and growth rate

**Fig. 1:**
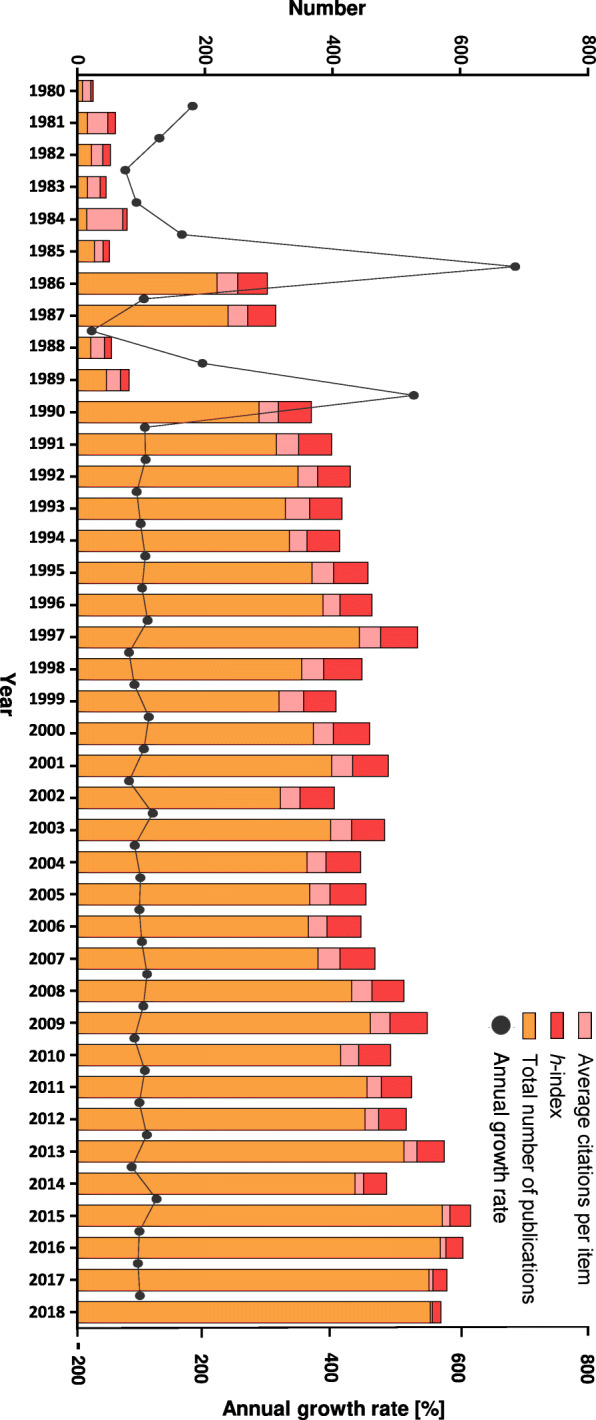
Overall publication performance in neuroblastoma research from 1980 to 2018, presented as a total number of publications per year, the corresponding annual growth rate, the average citations they received, and the associated *h*-index

We first assessed the overall publication performance in NB research during the last 38 years. In total, 12,435 documents, including 11,970 (96.2%) articles and 465 (9.8%) reviews, were published by 36,908 authors from 86 countries. The total publications output was very low prior to 1990 (*n* = 626, 5.0%) and began to increase extensively after 1991, reaching a peak in 2015 (*n* = 572, 4.6%). Linear fitting of the data revealed an increase in the number of publications written between 1980 and 2018 (r^2^ = 0.92 [CI: 0.86 to 0.96]; *p* < 0.0001]). The average annual percentage growth rate indicating increasing annual scientific production was 11.8%. The highest annual growth rates were noted in 1986 (711%) and in 1990 (519.5%) while the lowest was recorded in 1998 (− 91.1%). After 1991, the growth rates were stable, ranging from − 20.3 to + 31.5% (Fig. 1, Table S1).

### Citation rate and dynamics

**Fig. 2:**
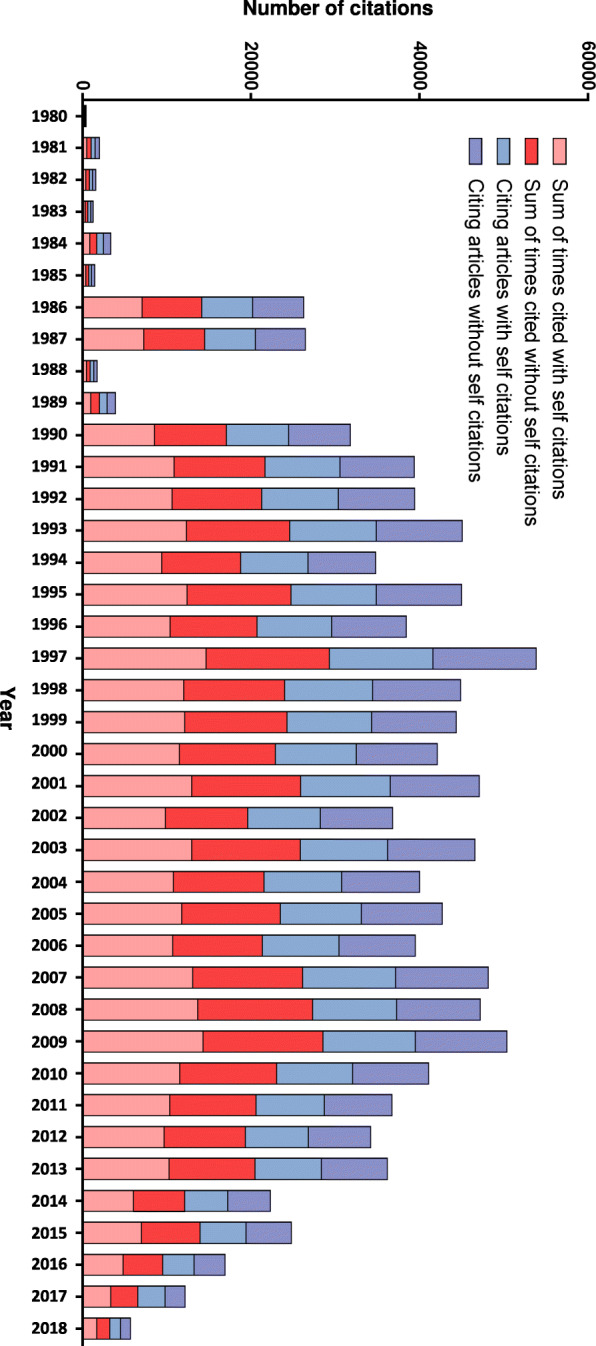
Detailed profile of citation rate and dynamics on neuroblastoma during the last 38 years

Of 12,136 retrieved documents, a total of 316,017 received citations including self-citations and 289,357 were without self-citations. The average citation per item (CPI) was 28.35 ± 7.7. There was a consistent citation dynamic ranging from 29.5 CPI in 1980 to 30.8 CPI in 2010. After 2011, the CPI was 12.7, which was lower compared to the period 1980–2010, because most newly published articles had not been cited much at the time of data extraction for our study. While the number of single-authored documents remains stable over time (r^2^ = − 0.6, *p* = 0.24), the number of multi-authored documents increased significantly (r^2^ = 1.0, *p* = 0.003) (Fig. 2, Table S2).

### Most prolific authors

In the entire dataset of 36,908 authors, 25,873 authors (70.1%) published a single paper related to neuroblastoma and were considered “occasional” authors; 5178 (14.0%) published two papers; 2076 (5.6%) published three papers; 3781 (10.2%) published four or more papers. Authors who published more than one paper were considered to be “core” authors. Of the top ten contributing authors, Berthold F (University of Cologne, Department of Pediatric Oncology and Hematology, University of Cologne, Koeln, Germany) was ranked first in the number of published articles (*n* = 169), Matthay KK (Department of Pediatrics and Helen Diller Family Comprehensive Cancer Center, University of California, San Francisco, California, USA) had the highest *h*- and *m*-indices (63 and 2.2, respectively) while Seeger RC (Division of Hematology/Oncology, Children’s Hospital Los Angeles, Los Angeles, USA) had the highest average citation per item count (100.2) (Table 1). Scientific productivity of the top authors on NB research over time is presented in Figure S1.

**Table 1:** Top 10 contributing authors in field of neuroblastoma research

Rank	Author	Number of publications	H index	G Index	M Index	Articles Fractionalized	Average citation per item	Sum of time cited ( without self citation)	PY start
1	Berthold F	169	45	80	1.3	27.6	43.4	7420 (6838)	1986
2	Matthay KK	165	63	113	2.2	21.4	81.0	13,373 (12,305)	1992
3	Cheung NKV	160	50	76	1.4	33.4	42.53	6975 (61645)	1986
4	Maris JM	158	56	110	2.2	19.0	79.8	12,661 (11,878)	1995
5	Cohn SL	156	54	96	1.6	20.4	64.2	10,016 (9434)	1987
6	Nakagawara A	118	33	67	0.9	17.8	40.9	4954 (4685)	1986
7	Seeger RC	116	57	108	1.6	–	100.2	11,725 (11,368)	1986
8	Speleman F	116	39	72	1.5	–	48.4	5614 (5132)	1994
9	Tonini GP	116	27	54	0.8	18.4	29.3	3429 (3163)	1987
10	Hero B	112	39	68	1.5	–	44.7	5014 (4673)	1995

Affiliation of the highly cited authors

Berthold F: University of Cologne, Department of Pediatric Oncology and Hematology, University of Cologne, Koeln, Germany

Matthay KK: Department of Pediatrics and Helen Diller Family Comprehensive Cancer Center, University of California, San Francisco, California, USA

Cheung NKV: Department of Pediatrics, Memorial Sloan-Kettering Cancer Center, New York, NY 10065, USA

Maris JM: The Children’s Hospital of Philadelphia, Division of Oncology, Philadelphia, USA

Cohn SL: Section of Hematology/Oncology, Department of Pediatrics, University of Chicago, Chicago

Nakagawara A: Saga Medical Center Koseikan, Saga, Japan

Seeger RC: Division of Hematology/Oncology, Children’s Hospital Los Angeles, Los Angeles, USA

Speleman F: Center for Medical Genetics Ghent, Medical Research Building 1, Ghent, Belgium

Tonini GP: Neuroblastoma Laboratory, Italian Neuroblastoma Foundation, Pediatric Research Institute, Fondazione Città della Speranza, Padua, Italy

Hero B: University of Cologne, Department of Pediatric Oncology and Hematology, University of Cologne, Koeln, Germany

### Core journals

In the time frame analyzed, there were 1828 academic journals publishing papers related to neuroblastoma research. *Journal of Neurochemistry* had the highest publication output (*n* = 319, 17.4%), followed by *Cancer Research* (*n* = 295, 16.1%), *Journal of Pediatric Surgery* (*n* = 278, 15.2%), and *Pediatric Blood and Cancer* (*n* = 261, 14.3%). The most cited journals were *Cancer Research* (*n* = 19,170), *Journal of Clinical Oncology* (*n* = 16,426), *Journal of Neurochemistry* (*n* = 10,221), *Oncogene* (*n* = 9223), and *Journal of Biological Chemistry* (*n* = 9197). *Cancer Research* (80) had the highest *h* index, following by *Journal of Clinical Oncology* (75), and *Journal of Biological Chemistry* (56). Table 2 summarized source impact of the top 20 journals publishing on NB.

**Table 2:** Source impact of the top journals publishing on neuroblastoma

Source	IF	NP	TC	***h***-index	***g***-index	***m***-index
Journal of Neurochemistry	4.87	319	10,221	53	71	1.39
Cancer Research	9.13	295	19,170	80	117	2.35
Journal of Pediatric Surgery	2.09	278	4409	33	45	0.86
Pediatric Blood and Cancer	2.64	261	3368	27	40	1.68
Oncogene	6.85	194	9223	55	82	1.83
PLOS One	2.77	193	3528	27	43	2.07
Neuroscience Letters	2.15	192	3568	31	45	0.86
Journal of Clinical Oncology	26.3	180	16,426	75	121	2.20
European Journal of Cancer	7.19	178	5229	40	57	1.37
International Journal of Cancer	7.3	172	5123	35	74	1.22
Cancer Letters	6.5	171	4066	35	48	1.12
Clinical Cancer Research	10.2	168	7228	49	70	1.96
Biochemical and Biophysical Research Communications	2.7	161	3517	32	48	0.78
Cancer	6.1	153	6935	46	76	1.15
Journal of Biological Chemistry	4.1	151	9197	56	87	1.4
Journal of Pediatric Hematology Oncology	0.9	146	1890	25	38	0.96
Brain Research	2.9	137	3390	30	48	0.83
Oncotarget	5.1	137	1740	23	30	2.3
British Journal of Cancer	5.9	117	3684	35	52	0.89

### Active countries

Eighty-six countries were involved in NB total research output. Among them, 9999 (80.4%) of publications were contributed by the top twelve most productive countries, putting out more than 300 publications (Table 3). The United States of America (USA) published the most papers (*n* = 4328), had the highest *h*-index (141), and ranked first in terms of single country publications (*n* = 2284). Other high prolific countries were Japan (*n* = 1364), Italy (*n* = 1336), and Germany (*n* = 1128). The Netherlands had the highest rate of average article citations (*n* = 39.12), followed by the USA (*n* = 35.45), France (*n* = 33.24), Sweden (*n* = 33.16), and China (*n* = 32.61).

**Table 3:** Most productive countries contributing to neuroblastoma research

Region	TP	***h***-index	TC	AAC
USA	4328	141	113,525	35.45
Japan	1364	76	23,268	24.66
Italy	1336	78	26,134	27.85
Germany	1128	85	21,597	31.19
UK	910	75	18,424	26.78
China	829	44	9144	32.61
France	748	70	14,846	33.24
Sweden	454	56	10,149	33.16
Spain	454	55	7610	30.00
Canada	427	52	7468	27.21
Netherlands	337	54	13,184	39.12
South Korea	330	34	5269	15.97

*TP* total production, *TC* total citations, *AAC* average article citations

### International collaborations

Researchers from the USA showed the highest collaboration performance with a total link strength (TLS) of 1438, followed by Germany (TLS = 852), the United Kingdom (TLS = 829), Italy (TLS = 801), and France (TLS = 707). International collaboration analysis showed that 136 articles (30.0%) produced by Sweden had international authors, followed by authors from the UK (*n* = 221, 24.3%), France (*n* = 167, 22.3%), Germany (*n* = 244, 21.6%), and the USA (*n* = 918, 21.2%). The international collaboration network is presented in Figure S2. The number of links between any two countries represents the strength of collaboration, while the color intensity is proportional to the number of publications. The strongest collaboration was between the USA and Germany (frequency, *n* = 160), the USA and Italy (*n* = 156), the USA and the UK (*n* = 137), and the UK and Italy (*n* = 131).

### Most cited NB papers and NB papers without a single citation

Of 12,435 publications related to NB, 12,136 (94.8%) were cited at least one time and 299 (2.4%) publications remain uncited after their publication. Table 4 demonstrates the top ten studies according to total number of citations. The review article entitled “Revisions of the international criteria for neuroblastoma diagnosis, staging, and response to treatment” published by Broder GM in *Journal of Clinical Oncology* in 1993 received the highest number of citations (*n* = 1450).

**Table 4:** Most cited neuroblastoma papers

Authors^**a**^	Article	Journal	Year	Vol	Issue	Page	TC	TC per year
Broder GM, Pritchard J, Berthold F, Hedborg F	Revisions of the international criteria for neuroblastoma diagnosis, staging, and response to treatment.	J Clin Oncol	1993	11	8	1466–77	1450	55.7
Kaghad M,Bonnet H, Yang A, Caput D	Monoallelically expressed gene related to p53 at 1p36, a region frequently deleted in neuroblastoma and other human cancers	Cell	1997	90	4	809–19	1403	63.7
Broder GM	Neuroblastoma: Biological insights into a clinical enigma	Nat Rec Cancer	2003	3	3	203–16	1328	83.0
Matthay KK, Villablanca JG, Seeger RC, Reynolds	Treatment of high-risk neuroblastoma with intensive chemotherapy, radiotherapy, autologous bone marrow transplantation, and 13-cis-retinoic acid	N Engl J Med	1999	341	16	1165–73	1246	62.3
Maris JM, Hogarty MD, Bagatell R, Cohn SL	Neuroblastoma	Lancet	2010	369	9579	2106–20	1153	96.1
Maris JM	Recent Advances in Neuroblastoma	N Engl J Med	2010	362	23	2202–11	792	88.0
Yu AL, Gilman AL, Ozkaynak MF, Sondel PM	Anti-GD2 Antibody with GM-CSF, Interleukin-2, and Isotretinoin for Neuroblastoma	N Engl J Med	2010	363	14	1324–34	707	78.5
Mosse YP, Laudenslager M, Longo L, Maris JM	Identification of ALK as a major familial neuroblastoma predisposition gene	Nature	2008	455	7215	930–5	704	64.0
Shimada H, Chatten J, Newton WA, Misugi K	Histopathologic prognostic factors in neuroblastic tumors: definition of subtypes of ganglioneuroblastoma and an age-linked classification of neuroblastomas	J Natl Cancer Inst	1984	73	2	405–16	686	19.6
Pule M, Savoldo B, Myers GD, Brenner MK	Virus-specific T cells engineered to coexpress tumor-specific receptors: persistence and antitumor activity in individuals with neuroblastoma	Nat Med	2008	14	11	1264–70	674	61.3

^**a**^ first, second, third, and last authors

### Keywords analysis

**Fig. 3:**
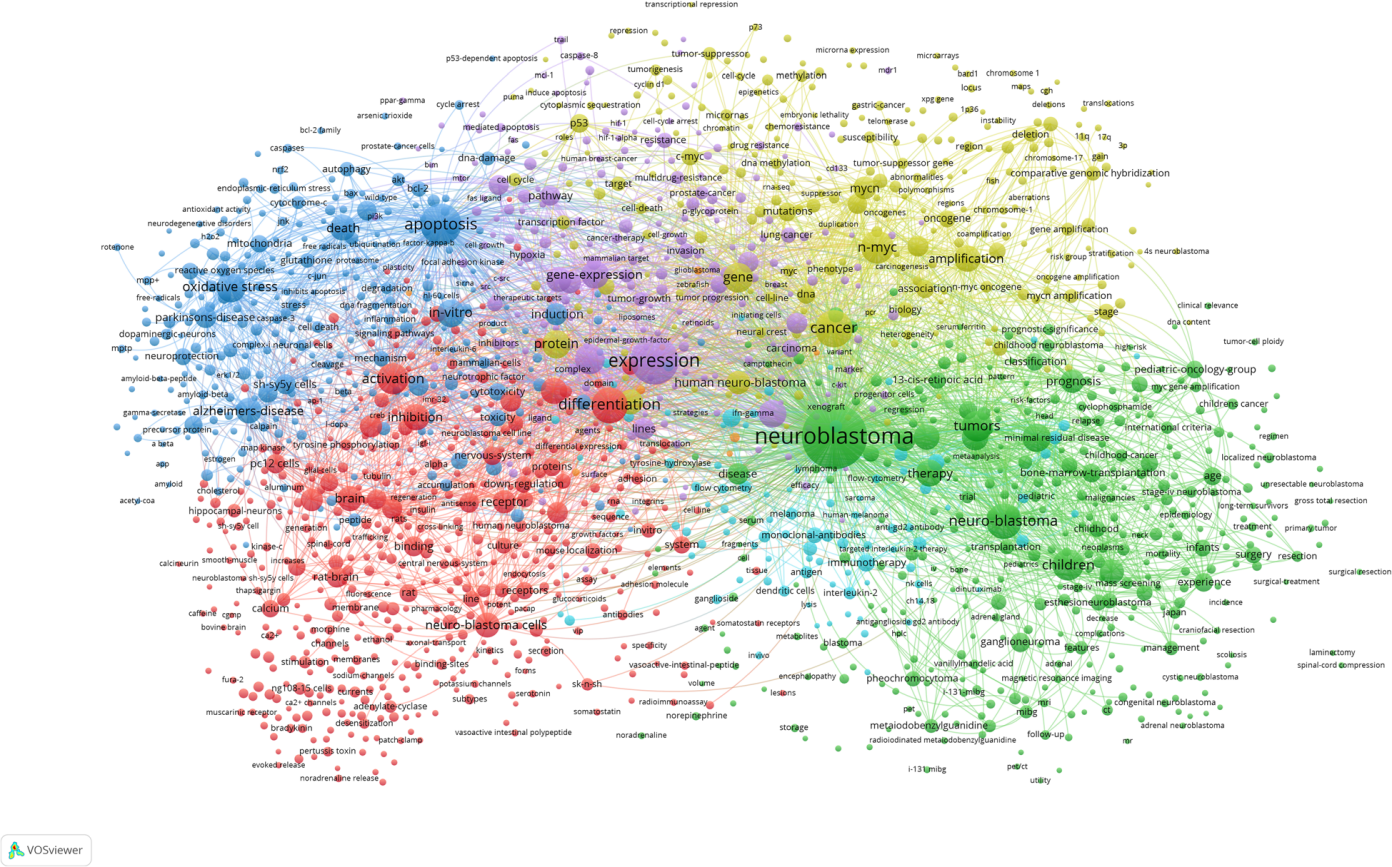
The keywords co-occurrence network. Minimum number of occurrence of a keyword = 10, minimum links strength = 10. Overall, 1638 keywords met threshold criteria. There are 7 clusters of keywords: red indicates Cluster 1 (*n* = 449), green indicates Cluster 2 (*n* = 341), blue indicates Cluster 3 (*n* = 285), yellow indicates Cluster 4 (*n* = 246), purple indicates Cluster 5 (*n* = 214), light-blue indicates Cluster 6 (*n* = 94), orange indicates Cluster 7 (*n* = 9)

The most frequent author’s keywords were “neuroblastoma” (*n* = 4505), “apoptosis” (*n* = 821), “differentiation” (*n* = 371), “mycn” (*n* = 262), “ganglioneuroma” (*n* = 222), “oxidative stress” (*n* = 218), “neuroblastoma cells” (*n* = 214), “retinoic acid” (*n* = 195), “chemotherapy” (*n* = 153), “SH-SY5Y” (*n* = 153). The overall keyword network visualization is presented in Fig. 3. We identified keywords with a high-citation burst, which can be used to predict research areas attracting an extraordinary degree of attention (Figure S3). Next, we aimed to map the conceptual co-word structure using the word co-occurrences in our bibliographic metadata to identify clusters of documents which express common concepts. The results are plotted on a two-dimensional map (Figure S4). Overall, 7 clusters of words could be identified (each color represents a cluster of word). The three-fields plot shows the relationship between the author’s keywords (research contents = right field), references authors use (intelectual roots = left field), and the top authors (middle field) (Figure S5).

## Discussion

In this scientometric study, we demonstrated the overall NB research output during the last 38 years, with the total number of publications reaching 12,435 articles in 2018. Overall, the number of NB-related papers has increased 69-fold since the 1980s, probably reflecting the biological and clinical heterogeneity as well as the diversity of NB research sub-fields. We also showed the average annual percentage growth rate of 11.8%. This rate was higher than that for both cancer research as a whole (6.5%) and global pediatric cancer research, (4.3%) indicating high scientific interest in NB research [25, 26]. We detected an extensive increase in number of publications and corresponding growth rate of NB papers after 1991, which may reflect the concentrated research to establish international criteria for NB diagnosis, staging, and treatment strategies [27–29]. Regarding the number of publications as a proxy for quantity of research, it is difficult to make direct comparisons to other pediatric and non-pediatric oncological scientometric studies, as the time periods of investigation vary significantly and research areas are represented differently in the literature [30]. For instance, as recently shown by Syrimi et al., pediatric leukemia and tumors of central nervous system were the most dominant research areas during 2007–2016, which may reflect the prevalence of these diseases [26].

Performance indicators measured by the number of received citations are used to identify the quality of the scientific publication and gauge its impact on the scientific community [31]. In our study, retrieved documents received a total of 316,017 citations with an average citation per document of 28.35 ± 7.7. This was higher than for other rare oncological diseases, such as male breast cancer with a total number of 76,104 citations [32], but lower compared to more prevalent cancers, such as female breast cancer (*n* = 4,136,224 citations) [33].

We showed that 12 (11.6%) of 86 countries were responsible for 80% of NB-related research output. Of these, the USA was the leading country regarding total number of publications, *h*-index, total citations, and average article citations. As a high-income country, the USA allocates a large budget to research and has a vast number of research centers [34–36].

There is a global trend in science towards national and international collaborations to improve patient care [37–39]. Especially for NB as a rare and highly complex oncological disease, international collaboration and the pooling of data is essential for conducting clinical trials of high statistical power. We were able to demonstrate that the USA had the highest collaboration performance, especially with Germany, Italy, and the UK.

Among the top 20 journals publishing articles on NB, 13 (65%) were listed in the category “Oncology” while the remaining 7 (35%) constituted distinct categories such as “Surgery” (*n* = 1), “Neurosciences” (*n* = 3), “Biochemistry Molecular Biology” (*n* = 2), “Multidisciplinary Sciences” (*n* = 1). The frequent publishing of NB-related papers indicates that the interest of readers and journal editors in *Journal of Neurochemistry*, *Journal of Pediatric Surgery*, *PLOS One* and *Neuroscience Letters* was also very high. Moreover, the *Journal of Neurochemistry* published the highest number of NB related articles, indicating the high significance of the molecular, cellular and biochemical aspects of NB research.

The most cited paper was the conference-related paper written by Broder GM, containing modifications to and clarifications of the International Neuroblastoma Staging System (INSS) and International Neuroblastoma Response Criteria (INRC). An additional three out of the ten most-cited articles were directly linked with the molecular and genetic factors involved in NB tumorigenesis. The identification of these tumor features and consequent discovery of druggable targets, such as ganglioside GD2 antibodies, has led to improvement of clinical outcomes [40]. Another four papers were excellent review/seminar articles focusing predominantly on tumor biology. These reported on the potential for novel targeted treatment options, particularly monoclonal antibodies [41]. However, among the 465 (9.8%) review articles included in our bibliographic dataset, many excellent papers were not included in the top-ten list. This phenomenon is known as the “Matthew Effect”: highly cited papers, scientists, and journals are cited more frequently than those with few citations [42].

The keywords employed most often by authors reflect the dynamics of research hotspots during the study period. We found that the keywords “neuroblastoma” and “apoptosis” were the most common and showed the greatest increase over time. Additionally, all of the top keywords with the strongest citation burst were related to the molecular-biological topics in NB research, suggesting the high significance of this NB sub-field. However, the examination of the field’s conceptual structure through a co-word analysis revealed other thematic network clusters, indicating diversity within research sub-fields.

Some limitations of our study should be addressed in future scientometric research. First, we used only the Web of Science™ database to search for publications, neglecting other search engines such as Scopus, Google Scholar or Index Medicus. Thus, other sources may yield different numbers of research items or citation counts. Second, due to constantly changing citation volumes over time, the results of our study are of temporary nature and valid for the time point of the present study’s data extraction (November 12, 2019). Third, the share of non-cited papers should also be considered when determining the *h*-index and impact factor of the author, article, journal and country. Nevertheless, we believe that our study provides a detailed scientometric analysis and improves insights into international research on NB.

## Conclusions

This scientometric study provides an in-depth analysis of global neuroblastoma research, highlighting the multidisciplinary nature of the NB community. Over the past four decades, NB research has progressed enormously, resulting in a better understanding of underlying tumor biology and leading to the development of new molecular therapies. Collaborative research has led to substantial progress in patient stratification and implementation of standardized treatment protocols. Studies like this one are useful for researchers, clinicians, journal editors, and others working on NB in order to understand the strengths and potential gaps in the research and to plan future investments in data collection and science policy. Given the disease burden, especially associated with high-risk NB, a specific analysis of research publications and collaboration networks in this area is warranted to build on the more general scientometric studies.

## Supplementary information

**Additional file 1 Figure S1** Individual profiling of the top ten authors with regard to the number of published articles and total citations (TC) received per year. The size of the circles indicate the number of publications per year.

**Additional file 2 Figure S2** A choropleth map detailing the geographic distribution of collaborating countries. The color intensity (from light-blue to dark-blue) is proportional to the number of publications. The number of links (presented as red lines) between any two countries represents the strength of collaboration.

**Additional file 3 Figure S3** Top 10 keywords with the strongest citation bursts during last 38 years

**Additional file 4 Figure S4** Common conceptual frames associated with neuroblastoma studies. Clustering of the 12,435 retrieved articles, including 7 different concepts of clusters of sizes 8, 5, 5, 11, 6, and 2 reflecting concepts frequently linked to neuroblastoma research.

**Additional file 5 Figure S5** The three-fields plot shows the relationship between the author’s keywords (research contents = right field), references authors use (intellectual roots = left field), and the top authors (middle field).

**Additional file 6 Table S1:** Total number of publications with corresponding average citations per item, *h*-index, and annual growth rate

**Additional file 7 Table S2:** Citation rate and dynamics

## Data Availability

The datasets used and analyzed in this study are available from the corresponding author on reasonable request.

## Abbreviations

NB: Neuroblastoma
GNB: Ganglioneuroblastoma
GN: Ganglioneuroma
MYCN: N-myc proto-oncogene protein
ALK: Anaplastic lymphoma kinase
CAC: Corresponding author’s country
SCP: Single country publication
MCP: Multiple countries publications
TC: Total citations
AAC: Average article citations
*h*-index: Hirsch index
CI: Collaboration index
MCA: Multiple Correspondence Analysis

## References

[1] Matthay KK, Maris JM, Schleiermacher G, Nakagawara A, Mackall CL, Diller L (2016). Neuroblastoma. Nat Rev Dis Primers.

[2] Esiashvili N, Anderson C, Katzenstein HM (2009). Neuroblastoma. Curr Probl Cancer.

[3] Maris JM (2010). Recent advances in neuroblastoma. N Engl J Med.

[4] Smith MA, Seibel NL, Altekruse SF, Ries LA, Melbert DL, O'Leary M (2010). Outcomes for children and adolescents with cancer: challenges for the twenty-first century. J Clin Oncol.

[5] Spix C, Pastore G, Sankila R, Stiller CA, Steliarova-Foucher E (2006). Neuroblastoma incidence and survival in European children (1978–1997): report from the Automated Childhood Cancer Information System project. Eur J Cancer (Oxford, England : 1990).

[6] Decarolis B, Simon T, Krug B, Leuschner I, Vokuhl C, Kaatsch P (2016). Treatment and outcome of Ganglioneuroma and Ganglioneuroblastoma intermixed. BMC Cancer.

[7] Brodeur GM (2003). Neuroblastoma: biological insights into a clinical enigma. Nat Rev Cancer.

[8] Johnsen JI, Dyberg C, Fransson S, Wickstrom M (2018). Molecular mechanisms and therapeutic targets in neuroblastoma. Pharmacol Res.

[9] Lee JW, Son MH, Cho HW, Ma YE, Yoo KH, Sung KW (2018). Clinical significance of MYCN amplification in patients with high-risk neuroblastoma. Pediatr Blood Cancer.

[10] Valentijn LJ, Koster J, Haneveld F, Aissa RA, van Sluis P, Broekmans ME (2012). Functional MYCN signature predicts outcome of neuroblastoma irrespective of MYCN amplification. Proc Natl Acad Sci U S A.

[11] Trigg Ricky, Turner Suzanne (2018). ALK in Neuroblastoma: Biological and Therapeutic Implications. Cancers.

[12] Bresler SC, Weiser DA, Huwe PJ, Park JH, Krytska K, Ryles H (2014). ALK mutations confer differential oncogenic activation and sensitivity to ALK inhibition therapy in neuroblastoma. Cancer Cell.

[13] Van Arendonk Kyle, Chung Dai (2019). Neuroblastoma: Tumor Biology and Its Implications for Staging and Treatment. Children.

[14] Whittle SB, Smith V, Doherty E, Zhao S, McCarty S, Zage PE (2017). Overview and recent advances in the treatment of neuroblastoma. Expert Rev Anticancer Ther.

[15] Modak S, Cheung NK (2010). Neuroblastoma: therapeutic strategies for a clinical enigma. Cancer Treat Rev.

[16] Park JR, Bagatell R, London WB, Maris JM, Cohn SL, Mattay KK (2013). Children's oncology Group's 2013 blueprint for research: neuroblastoma. Pediatr Blood Cancer.

[17] Pinto NR, Applebaum MA, Volchenboum SL, Matthay KK, London WB, Ambros PF (2015). Advances in risk classification and treatment strategies for neuroblastoma. J Clin Oncol.

[18] Oeffinger KC, Mertens AC, Sklar CA, Kawashima T, Hudson MM, Meadows AT (2006). Chronic health conditions in adult survivors of childhood cancer. N Engl J Med.

[19] Laverdière C, Liu Q, Yasui Y, Nathan PC, Gurney JG, Stovall M (2009). Long-term outcomes in survivors of neuroblastoma: a report from the childhood Cancer survivor study. J Natl Cancer Inst.

[20] Nguyen R, Dyer MA. Chapter 3 - neuroblastoma: molecular mechanisms and therapeutic interventions. Columbia, SC, United States. In: Ray SK, editor. Neuroblastoma: Academic Press; 2019. p. 43–61.

[21] Aria M, Cuccurullo C (2017). Bibliometrix: an R-tool for comprehensive science mapping analysis. J Informetrics.

[22] Hirsch JE (2005). An index to quantify an individual's scientific research output. Proc Natl Acad Sci U S A.

[23] Greenacre MJ (1991). Interpreting multiple correspondence analysis. Appl Stochastic ModelsData Anal.

[24] van Eck NJ, Waltman L (2010). Software survey: VOSviewer, a computer program for bibliometric mapping. Scientometrics.

[25] Begum M, Lewison G, Lawler M, Sullivan R (2018). Mapping the European cancer research landscape: An evidence base for national and Pan-European research and funding. Eur J Cancer (Oxford, England : 1990).

[26] Syrimi E, Lewison G, Sullivan R, Kearns P (2020). Analysis of global pediatric Cancer research and publications. JCO Global Oncol.

[27] Brodeur GM, Pritchard J, Berthold F, Carlsen NL, Castel V, Castelberry RP (1993). Revisions of the international criteria for neuroblastoma diagnosis, staging, and response to treatment. J Clin Oncol.

[28] Look AT, Hayes FA, Shuster JJ, Douglass EC, Castleberry RP, Bowman LC (1991). Clinical relevance of tumor cell ploidy and N-myc gene amplification in childhood neuroblastoma: a pediatric oncology group study. J Clin Oncol.

[29] Layfield LJ, Thompson JK, Dodge RK, Kerns BJ (1995). Prognostic indicators for neuroblastoma: stage, grade, DNA ploidy, MIB-1-proliferation index, p53, HER-2/neu and EGFr--a survival study. J Surg Oncol.

[30] Cabral BP, da Graca Derengowski Fonseca M, Mota FB (2018). The recent landscape of cancer research worldwide: a bibliometric and network analysis. Oncotarget.

[31] Finch A. 10 - citation, bibliometrics and quality: assessing impact and usage. In: Campbell R, Pentz E, Borthwick I, editors. Oxford: Academic and Professional Publishing: Chandos Publishing; 2012. p. 243–67.

[32] Dwivedi S, Garg KC, Prasad NH (2017). Scientometric profile of global male breast cancer research. Curr Sci.

[33] Glynn RW, Scutaru C, Kerin MJ, Sweeney KJ (2010). Breast cancer research output, 1945-2008: a bibliometric and density-equalizing analysis. Breast Cancer Res.

[34] Flotte TR (2017). The science policy implications of a trump presidency. Hum Gene Ther.

[35] Gostin LO (2009). Government and science: the unitary executive versus freedom of scientific inquiry. Hast Cent Rep.

[36] Groneberg-Kloft B, Scutaru C, Kreiter C, Kolzow S, Fischer A, Quarcoo D (2008). Institutional operating figures in basic and applied sciences: scientometric analysis of quantitative output benchmarking. Health Res Policy Syst.

[37] Greene M (2007). The demise of the lone author. Nature.

[38] Rosson NJ, Hassoun HT (2017). Global collaborative healthcare: assessing the resource requirements at a leading Academic Medical Center. Glob Health.

[39] Butrous G (2008). International cooperation to promote advances in medicine. Ann Thorac Med.

[40] Cheung NK, Dyer MA (2013). Neuroblastoma: developmental biology, cancer genomics and immunotherapy. Nat Rev Cancer.

[41] Yu AL, Gilman AL, Ozkaynak MF, London WB, Kreissman SG, Chen HX (2010). Anti-GD2 antibody with GM-CSF, interleukin-2, and isotretinoin for neuroblastoma. N Engl J Med.

[42] Merton R. K. (1968). The Matthew Effect in Science: The reward and communication systems of science are considered. Science.

