# Supplementary figures and images for: A scientometric analysis of neuroblastoma research
Source: BMC Cancer. 2020 May 29;20:486. doi: 10.1186/s12885-020-06974-3 (PMC7260742; doi:10.1186/s12885-020-06974-3)

## Top author's production of time

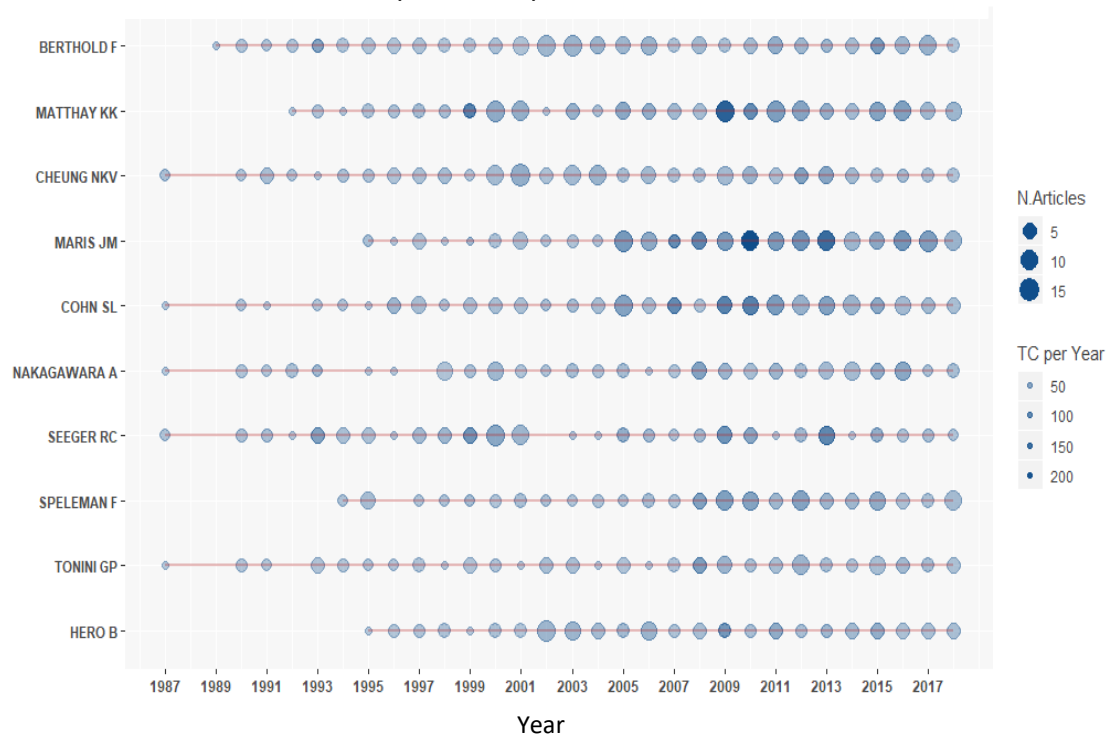

Supplement: Supplementary file 1 — Additional file 1 Figure S1 Individual profiling of the top ten authors with regard to the number of published articles and total citations (TC) received per year. The size of the circles indicate the number of publications per year. [file 12885_2020_6974_MOESM1_ESM.pdf]

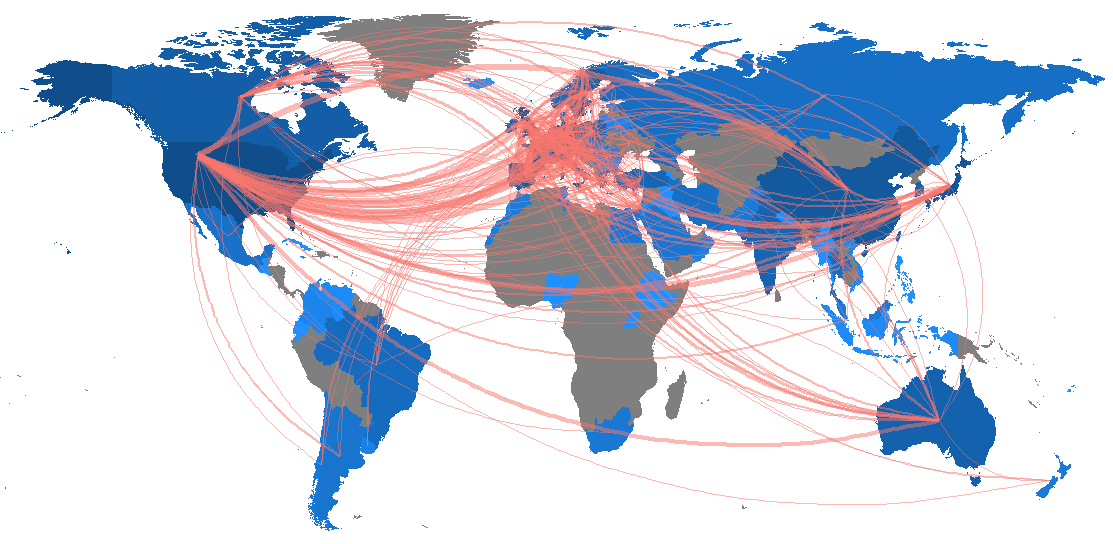

Supplement: Supplementary file 2 — Additional file 2 Figure S2 A choropleth map detailing the geographic distribution of collaborating countries. The color intensity (from light-blue to dark-blue) is proportional to the number of publications. The number of links (presented as red lines) between any two countries represents the strength of collaboration. [file 12885_2020_6974_MOESM2_ESM.png]

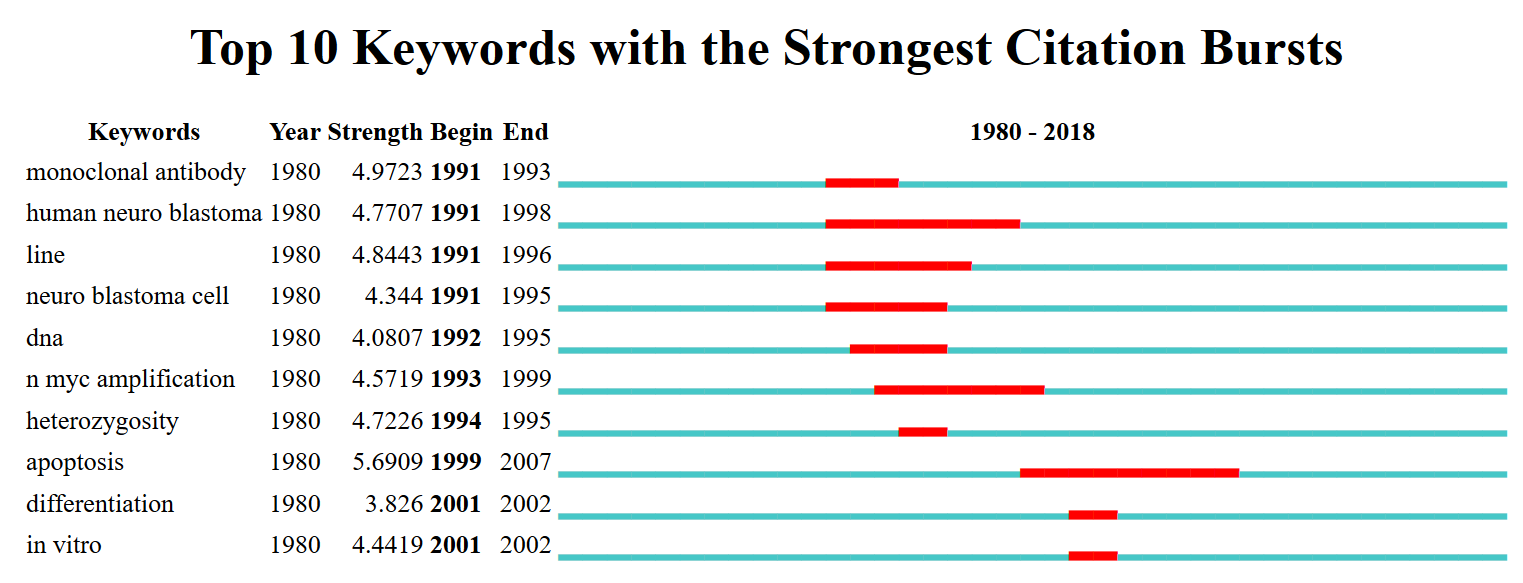

Supplement: Supplementary file 3 — Additional file 3 Figure S3 Top 10 keywords with the strongest citation bursts during last 38 years [file 12885_2020_6974_MOESM3_ESM.png]

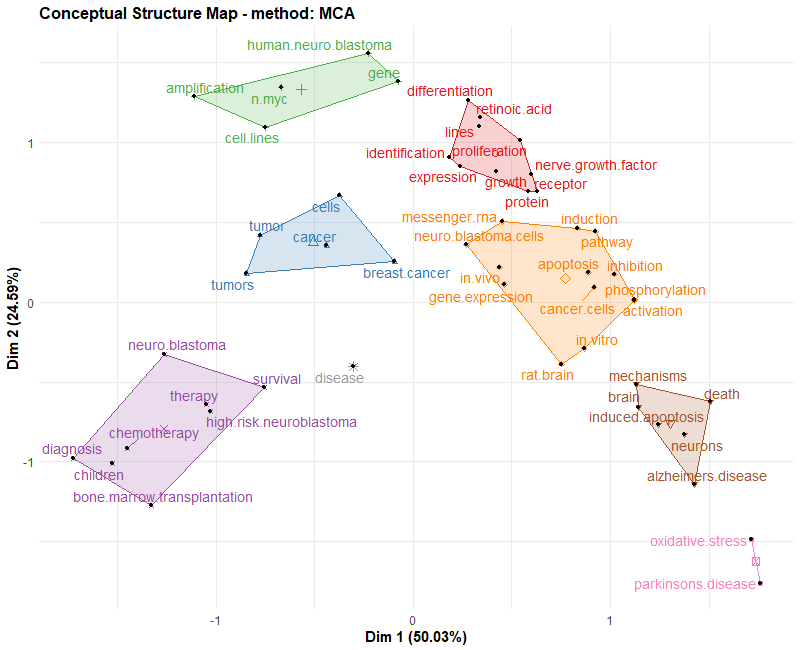

Supplement: Supplementary file 4 — Additional file 4 Figure S4 Common conceptual frames associated with neuroblastoma studies. Clustering of the 12,435 retrieved articles, including 7 different concepts of clusters of sizes 8, 5, 5, 11, 6, and 2 reflecting concepts frequently linked to neuroblastoma research. [file 12885_2020_6974_MOESM4_ESM.png]

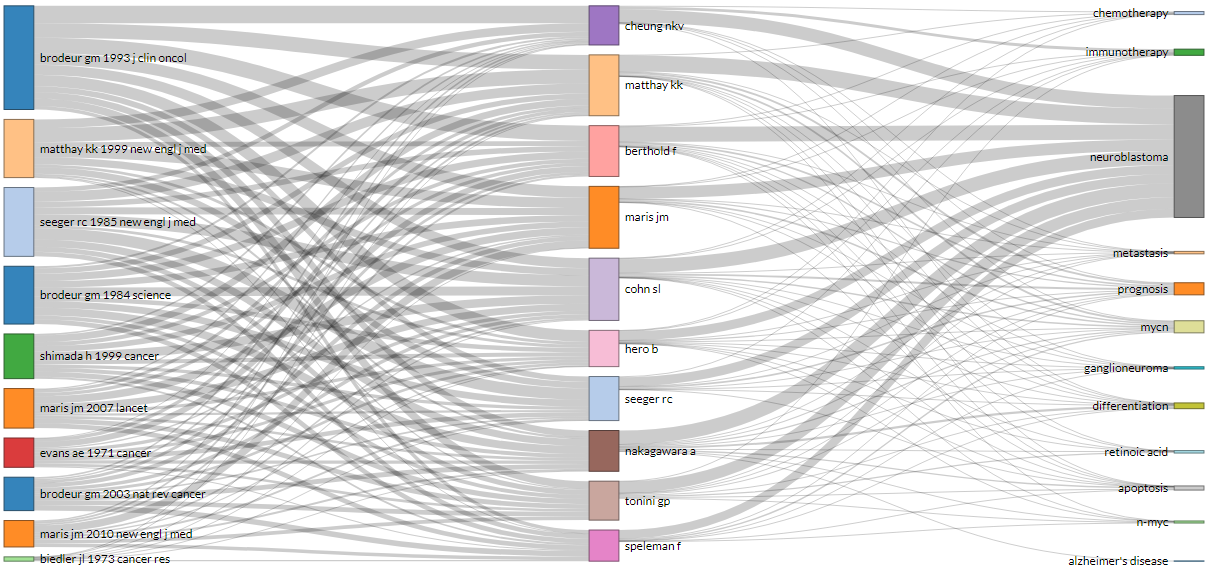

Supplement: Supplementary file 5 — Additional file 5 Figure S5 The three-fields plot shows the relationship between the author’s keywords (research contents = right field), references authors use (intellectual roots = left field), and the top authors (middle field). [file 12885_2020_6974_MOESM5_ESM.png]
