# Supplementary material for: A scientometric analysis of neuroblastoma research
Source: BMC Cancer. 2020 May 29;20:486. doi: 10.1186/s12885-020-06974-3 (PMC7260742; doi:10.1186/s12885-020-06974-3)
Supplement: Supplementary file 6 — Additional file 6 Table S1: Total number of publications with corresponding average citations per item, h-index, and annual growth rate [file 12885_2020_6974_MOESM6_ESM.docx]

| **Year** | **Total publications** | **Corresponding average citations per item** | ***h*-index** | **Annual growth rate (%)** |
| --- | --- | --- | --- | --- |
| **1980** | 8 | 13.25 | 4 |  |
| **1981** | 16 | 32 | 12 | + 100 |
| **1982** | 22 | 18.23 | 12 | + 37.5 |
| **1983** | 16 | 20.25 | 9 | - 27.3 |
| **1984** | 15 | 56.33 | 7 | - 6.25 |
| **1985** | 27 | 13.52 | 10 | + 80 |
| **1986** | 219 | 32.4 | 47 | + 711 |
| **1987** | 235 | 30.86 | 44 | + 7.76 |
| **1988** | 21 | 21.48 | 11 | - 91.1 |
| **1989** | 46 | 21.5 | 14 | + 119 |
| **1990** | 285 | 30.05 | 52 | + 519.5 |
| **1991** | 312 | 34.77 | 52 | + 9.47 |
| **1992** | [346](https://apps.webofknowledge.com/OutboundService.do?action=go&mode=raService&queryOption(summary_search_mode)=GeneralSearch&SID=C6XYSv4Y8ErSIZwNzgd&product=WOS&parentProduct=WOS&parentQid=60&colName=WOS&MARKEDLIST=) | 30.76 | 51 | + 10.9 |
| **1993** | 326 | 37.83 | 51 | - 5.8 |
| **1994** | 332 | 28.31 | 51 | + 1.84 |
| **1995** | 368 | 33.67 | 54 | + 10.8 |
| **1996** | 385 | 26.97 | 50 | + 4.61 |
| **1997** | 442 | 33.21 | 58 | + 14.8 |
| **1998** | 352 | 34.09 | 60 | - 20.3 |
| **1999** | 316 | 38.44 | 51 | - 10.2 |
| **2000** | 370 | 30.99 | 57 | + 17.1 |
| **2001** | 399 | 32.52 | 56 | + 7.83 |
| **2002** | 318 | 30.92 | 54 | - 20.3 |
| **2003** | 397 | 32.61 | 52 | + 24.8 |
| **2004** | 360 | 29.95 | 54 | - 9.3 |
| **2005** | 364 | 32.32 | 56 | + 1.1 |
| **2006** | 362 | 29.56 | 53 | - 0.5 |
| **2007** | 377 | 34.69 | 55 | + 4.14 |
| **2008** | 430 | 31.83 | 50 | + 14.1 |
| **2009** | 459 | 31.19 | 58 | + 6.74 |
| **2010** | 413 | 27.95 | 50 | - 10.1 |
| **2011** | 454 | 22.86 | 47 | + 9.93 |
| **2012** | 451 | 21.51 | 43 | - 0.6 |
| **2013** | 512 | 20.09 | 43 | + 13.5 |
| **2014** | 435 | 14 | 36 | - 15.1 |
| **2015** | 572 | 12.25 | 32 | + 31.5 |
| **2016** | 569 | 8.47 | 27 | - 0.5 |
| **2017** | 551 | 6.04 | 22 | - 3.2 |
| **2018** | 553 | 3.07 | 14 | + 0.18 |

**Table S1: Total number of publications with corresponding average citations per item, *h*-index, and annual growth rate**
