# Supplementary material for: A scientometric analysis of neuroblastoma research
Source: BMC Cancer. 2020 May 29;20:486. doi: 10.1186/s12885-020-06974-3 (PMC7260742; doi:10.1186/s12885-020-06974-3)
Supplement: Supplementary file 7 — Additional file 7 Table S2: Citation rate and dynamics [file 12885_2020_6974_MOESM7_ESM.docx]

| **Year** | **Sum of Times Cited with self citation** | **Sum of Times Cited without self citations** | **Citing articles with self citations** | **Citing articles without self citations** |
| --- | --- | --- | --- | --- |
| **1980** | 106 | 106 | [99](https://apps.webofknowledge.com/TotalCitingArticles.do?product=WOS&search_mode=TotalCitingArticles&qid=85&action=totalCA&SID=C6XYSv4Y8ErSIZwNzgd&viewType=summary&betterCount=99) | [99](https://apps.webofknowledge.com/TotalCitingArticles.do?product=WOS&search_mode=TotalCitingArticles&qid=85&action=totalCA&SID=C6XYSv4Y8ErSIZwNzgd&viewType=summary&betterCount=99) |
| **1981** | 512 | 512 | [486](https://apps.webofknowledge.com/TotalCitingArticles.do?product=WOS&search_mode=TotalCitingArticles&qid=83&action=totalCA&SID=C6XYSv4Y8ErSIZwNzgd&viewType=summary&betterCount=486) | [486](https://apps.webofknowledge.com/TotalCitingArticles.do?product=WOS&search_mode=TotalCitingArticles&qid=83&action=totalCA&SID=C6XYSv4Y8ErSIZwNzgd&viewType=summary&betterCount=486) |
| **1982** | 401 | 401 | [388](https://apps.webofknowledge.com/TotalCitingArticles.do?product=WOS&search_mode=TotalCitingArticles&qid=81&action=totalCA&SID=C6XYSv4Y8ErSIZwNzgd&viewType=summary&betterCount=388) | [388](https://apps.webofknowledge.com/TotalCitingArticles.do?product=WOS&search_mode=TotalCitingArticles&qid=81&action=totalCA&SID=C6XYSv4Y8ErSIZwNzgd&viewType=summary&betterCount=388) |
| **1983** | 324 | 322 | [308](https://apps.webofknowledge.com/TotalCitingArticles.do?product=WOS&search_mode=TotalCitingArticles&qid=79&action=totalCA&SID=C6XYSv4Y8ErSIZwNzgd&viewType=summary&betterCount=308) | [306](https://apps.webofknowledge.com/NonSelfCitingArticles.do?product=WOS&search_mode=NonSelfCitingTCA&qid=79&action=nonselfCA&SID=C6XYSv4Y8ErSIZwNzgd&viewType=summary&betterCount=306) |
| **1984** | 845 | 845 | [835](https://apps.webofknowledge.com/TotalCitingArticles.do?product=WOS&search_mode=TotalCitingArticles&qid=77&action=totalCA&SID=C6XYSv4Y8ErSIZwNzgd&viewType=summary&betterCount=835) | [835](https://apps.webofknowledge.com/TotalCitingArticles.do?product=WOS&search_mode=TotalCitingArticles&qid=77&action=totalCA&SID=C6XYSv4Y8ErSIZwNzgd&viewType=summary&betterCount=835) |
| **1985** | 365 | 365 | [364](https://apps.webofknowledge.com/TotalCitingArticles.do?product=WOS&search_mode=TotalCitingArticles&qid=75&action=totalCA&SID=C6XYSv4Y8ErSIZwNzgd&viewType=summary&betterCount=364&cacheurlFromRightClick=no) | [364](https://apps.webofknowledge.com/TotalCitingArticles.do?product=WOS&search_mode=TotalCitingArticles&qid=75&action=totalCA&SID=C6XYSv4Y8ErSIZwNzgd&viewType=summary&betterCount=364&cacheurlFromRightClick=no) |
| **1986** | 7,096 | 7,075 | [6,052](https://apps.webofknowledge.com/TotalCitingArticles.do?product=WOS&search_mode=TotalCitingArticles&qid=73&action=totalCA&SID=C6XYSv4Y8ErSIZwNzgd&viewType=summary&betterCount=6052) | [6,034](https://apps.webofknowledge.com/NonSelfCitingArticles.do?product=WOS&search_mode=NonSelfCitingTCA&qid=73&action=nonselfCA&SID=C6XYSv4Y8ErSIZwNzgd&viewType=summary&betterCount=6034) |
| **1987** | 7,283 | 7,259 | [5,961](https://apps.webofknowledge.com/TotalCitingArticles.do?product=WOS&search_mode=TotalCitingArticles&qid=71&action=totalCA&SID=C6XYSv4Y8ErSIZwNzgd&viewType=summary&betterCount=5961) | [5,939](https://apps.webofknowledge.com/NonSelfCitingArticles.do?product=WOS&search_mode=NonSelfCitingTCA&qid=71&action=nonselfCA&SID=C6XYSv4Y8ErSIZwNzgd&viewType=summary&betterCount=5939) |
| **1988** | 451 | 451 | [425](https://apps.webofknowledge.com/TotalCitingArticles.do?product=WOS&search_mode=TotalCitingArticles&qid=69&action=totalCA&SID=C6XYSv4Y8ErSIZwNzgd&viewType=summary&betterCount=425) | [425](https://apps.webofknowledge.com/TotalCitingArticles.do?product=WOS&search_mode=TotalCitingArticles&qid=69&action=totalCA&SID=C6XYSv4Y8ErSIZwNzgd&viewType=summary&betterCount=425) |
| **1989** | 989 | 989 | [953](https://apps.webofknowledge.com/TotalCitingArticles.do?product=WOS&search_mode=TotalCitingArticles&qid=67&action=totalCA&SID=C6XYSv4Y8ErSIZwNzgd&viewType=summary&betterCount=953) | [953](https://apps.webofknowledge.com/NonSelfCitingArticles.do?product=WOS&search_mode=NonSelfCitingTCA&qid=67&action=nonselfCA&SID=C6XYSv4Y8ErSIZwNzgd&viewType=summary&betterCount=953) |
| **1990** | 8,565 | 8,533 | [7,365](https://apps.webofknowledge.com/TotalCitingArticles.do?product=WOS&search_mode=TotalCitingArticles&qid=65&action=totalCA&SID=C6XYSv4Y8ErSIZwNzgd&viewType=summary&betterCount=7365) | [7,337](https://apps.webofknowledge.com/NonSelfCitingArticles.do?product=WOS&search_mode=NonSelfCitingTCA&qid=65&action=nonselfCA&SID=C6XYSv4Y8ErSIZwNzgd&viewType=summary&betterCount=7337) |
| **1991** | 10,847 | 10,816 | 8,868 | [8,840](https://apps.webofknowledge.com/NonSelfCitingArticles.do?product=WOS&search_mode=NonSelfCitingTCA&qid=63&action=nonselfCA&SID=C6XYSv4Y8ErSIZwNzgd&viewType=summary&betterCount=8840) |
| **1992** | 10,642 | 10,623 | [9,083](https://apps.webofknowledge.com/TotalCitingArticles.do?product=WOS&search_mode=TotalCitingArticles&qid=61&action=totalCA&SID=C6XYSv4Y8ErSIZwNzgd&viewType=summary&betterCount=9083) | [9,065](https://apps.webofknowledge.com/NonSelfCitingArticles.do?product=WOS&search_mode=NonSelfCitingTCA&qid=61&action=nonselfCA&SID=C6XYSv4Y8ErSIZwNzgd&viewType=summary&betterCount=9065) |
| **1993** | 12,331 | 12,296 | [10,227](https://apps.webofknowledge.com/TotalCitingArticles.do?product=WOS&search_mode=TotalCitingArticles&qid=59&action=totalCA&SID=C6XYSv4Y8ErSIZwNzgd&viewType=summary&betterCount=10227) | [10,201](https://apps.webofknowledge.com/NonSelfCitingArticles.do?product=WOS&search_mode=NonSelfCitingTCA&qid=59&action=nonselfCA&SID=C6XYSv4Y8ErSIZwNzgd&viewType=summary&betterCount=10201) |
| **1994** | 9,399 | 9,367 | 8,014 | [7,988](https://apps.webofknowledge.com/NonSelfCitingArticles.do?product=WOS&search_mode=NonSelfCitingTCA&qid=57&action=nonselfCA&SID=C6XYSv4Y8ErSIZwNzgd&viewType=summary&betterCount=7988) |
| **1995** | 12,389 | 12,340 | [10,150](https://apps.webofknowledge.com/TotalCitingArticles.do?product=WOS&search_mode=TotalCitingArticles&qid=55&action=totalCA&SID=C6XYSv4Y8ErSIZwNzgd&viewType=summary&betterCount=10150) | [10,119](https://apps.webofknowledge.com/NonSelfCitingArticles.do?product=WOS&search_mode=NonSelfCitingTCA&qid=55&action=nonselfCA&SID=C6XYSv4Y8ErSIZwNzgd&viewType=summary&betterCount=10119) |
| **1996** | 10,382 | 10,344 | [8,871](https://apps.webofknowledge.com/TotalCitingArticles.do?product=WOS&search_mode=TotalCitingArticles&qid=53&action=totalCA&SID=C6XYSv4Y8ErSIZwNzgd&viewType=summary&betterCount=8871) | [8,839](https://apps.webofknowledge.com/NonSelfCitingArticles.do?product=WOS&search_mode=NonSelfCitingTCA&qid=53&action=nonselfCA&SID=C6XYSv4Y8ErSIZwNzgd&viewType=summary&betterCount=8839) |
| **1997** | 14,680 | 14,640 | [12,280](https://apps.webofknowledge.com/TotalCitingArticles.do?product=WOS&search_mode=TotalCitingArticles&qid=51&action=totalCA&SID=C6XYSv4Y8ErSIZwNzgd&viewType=summary&betterCount=12280) | [12,247](https://apps.webofknowledge.com/NonSelfCitingArticles.do?product=WOS&search_mode=NonSelfCitingTCA&qid=51&action=nonselfCA&SID=C6XYSv4Y8ErSIZwNzgd&viewType=summary&betterCount=12247) |
| **1998** | 11,998 | 11,974 | [10,453](https://apps.webofknowledge.com/TotalCitingArticles.do?product=WOS&search_mode=TotalCitingArticles&qid=49&action=totalCA&SID=C6XYSv4Y8ErSIZwNzgd&viewType=summary&betterCount=10453) | [10,434](https://apps.webofknowledge.com/NonSelfCitingArticles.do?product=WOS&search_mode=NonSelfCitingTCA&qid=49&action=nonselfCA&SID=C6XYSv4Y8ErSIZwNzgd&viewType=summary&betterCount=10434) |
| **1999** | 12,147 | 12,131 | [10,048](https://apps.webofknowledge.com/TotalCitingArticles.do?product=WOS&search_mode=TotalCitingArticles&qid=47&action=totalCA&SID=C6XYSv4Y8ErSIZwNzgd&viewType=summary&betterCount=10048) | [10,034](https://apps.webofknowledge.com/NonSelfCitingArticles.do?product=WOS&search_mode=NonSelfCitingTCA&qid=47&action=nonselfCA&SID=C6XYSv4Y8ErSIZwNzgd&viewType=summary&betterCount=10034) |
| **2000** | 11,465 | 11,418 | [9,619](https://apps.webofknowledge.com/TotalCitingArticles.do?product=WOS&search_mode=TotalCitingArticles&qid=45&action=totalCA&SID=C6XYSv4Y8ErSIZwNzgd&viewType=summary&betterCount=9619) | [9,585](https://apps.webofknowledge.com/NonSelfCitingArticles.do?product=WOS&search_mode=NonSelfCitingTCA&qid=45&action=nonselfCA&SID=C6XYSv4Y8ErSIZwNzgd&viewType=summary&betterCount=9585) |
| **2001** | 12,976 | 12,926 | [10,619](https://apps.webofknowledge.com/TotalCitingArticles.do?product=WOS&search_mode=TotalCitingArticles&qid=43&action=totalCA&SID=C6XYSv4Y8ErSIZwNzgd&viewType=summary&betterCount=10619) | [10,579](https://apps.webofknowledge.com/NonSelfCitingArticles.do?product=WOS&search_mode=NonSelfCitingTCA&qid=43&action=nonselfCA&SID=C6XYSv4Y8ErSIZwNzgd&viewType=summary&betterCount=10579) |
| **2002** | 9,833 | 9,810 | [8,583](https://apps.webofknowledge.com/TotalCitingArticles.do?product=WOS&search_mode=TotalCitingArticles&qid=41&action=totalCA&SID=C6XYSv4Y8ErSIZwNzgd&viewType=summary&betterCount=8583) | [8,564](https://apps.webofknowledge.com/NonSelfCitingArticles.do?product=WOS&search_mode=NonSelfCitingTCA&qid=41&action=nonselfCA&SID=C6XYSv4Y8ErSIZwNzgd&viewType=summary&betterCount=8564) |
| **2003** | 12,946 | 12,920 | [10,351](https://apps.webofknowledge.com/TotalCitingArticles.do?product=WOS&search_mode=TotalCitingArticles&qid=39&action=totalCA&SID=C6XYSv4Y8ErSIZwNzgd&viewType=summary&betterCount=10351) | [10,327](https://apps.webofknowledge.com/NonSelfCitingArticles.do?product=WOS&search_mode=NonSelfCitingTCA&qid=39&action=nonselfCA&SID=C6XYSv4Y8ErSIZwNzgd&viewType=summary&betterCount=10327) |
| **2004** | 10,781 | 10,753 | [9,245](https://apps.webofknowledge.com/TotalCitingArticles.do?product=WOS&search_mode=TotalCitingArticles&qid=37&action=totalCA&SID=C6XYSv4Y8ErSIZwNzgd&viewType=summary&betterCount=9245) | [9,222](https://apps.webofknowledge.com/NonSelfCitingArticles.do?product=WOS&search_mode=NonSelfCitingTCA&qid=37&action=nonselfCA&SID=C6XYSv4Y8ErSIZwNzgd&viewType=summary&betterCount=9222) |
| **2005** | 11,765 | 11,729 | [9,614](https://apps.webofknowledge.com/TotalCitingArticles.do?product=WOS&search_mode=TotalCitingArticles&qid=33&action=totalCA&SID=C6XYSv4Y8ErSIZwNzgd&viewType=summary&betterCount=9614) | [9,586](https://apps.webofknowledge.com/NonSelfCitingArticles.do?product=WOS&search_mode=NonSelfCitingTCA&qid=33&action=nonselfCA&SID=C6XYSv4Y8ErSIZwNzgd&viewType=summary&betterCount=9586) |
| **2006** | 10,699 | 10,656 | [9,091](https://apps.webofknowledge.com/TotalCitingArticles.do?product=WOS&search_mode=TotalCitingArticles&qid=31&action=totalCA&SID=C6XYSv4Y8ErSIZwNzgd&viewType=summary&betterCount=9091) | [9,057](https://apps.webofknowledge.com/NonSelfCitingArticles.do?product=WOS&search_mode=NonSelfCitingTCA&qid=31&action=nonselfCA&SID=C6XYSv4Y8ErSIZwNzgd&viewType=summary&betterCount=9057) |
| **2007** | 13,080 | 13,044 | [11,029](https://apps.webofknowledge.com/TotalCitingArticles.do?product=WOS&search_mode=TotalCitingArticles&qid=29&action=totalCA&SID=C6XYSv4Y8ErSIZwNzgd&viewType=summary&betterCount=11029) | 11,006 |
| **2008** | 13,687 | 13,630 | [9,966](https://apps.webofknowledge.com/TotalCitingArticles.do?product=WOS&search_mode=TotalCitingArticles&qid=27&action=totalCA&SID=C6XYSv4Y8ErSIZwNzgd&viewType=summary&betterCount=9966) | 9,931 |
| **2009** | 14,318 | 14,211 | [10,958](https://apps.webofknowledge.com/TotalCitingArticles.do?product=WOS&search_mode=TotalCitingArticles&qid=25&action=totalCA&SID=C6XYSv4Y8ErSIZwNzgd&viewType=summary&betterCount=10958) | [10,902](https://apps.webofknowledge.com/NonSelfCitingArticles.do?product=WOS&search_mode=NonSelfCitingTCA&qid=25&action=nonselfCA&SID=C6XYSv4Y8ErSIZwNzgd&viewType=summary&betterCount=10902) |
| **2010** | 11,545 | 11,495 | [9,022](https://apps.webofknowledge.com/TotalCitingArticles.do?product=WOS&search_mode=TotalCitingArticles&qid=23&action=totalCA&SID=C6XYSv4Y8ErSIZwNzgd&viewType=summary&betterCount=9022) | [8,991](https://apps.webofknowledge.com/NonSelfCitingArticles.do?product=WOS&search_mode=NonSelfCitingTCA&qid=23&action=nonselfCA&SID=C6XYSv4Y8ErSIZwNzgd&viewType=summary&betterCount=8991) |
| **2011** | 10,377 | 10,253 | [8,090](https://apps.webofknowledge.com/TotalCitingArticles.do?product=WOS&search_mode=TotalCitingArticles&qid=21&action=totalCA&SID=C6XYSv4Y8ErSIZwNzgd&viewType=summary&betterCount=8090) | [8,036](https://apps.webofknowledge.com/NonSelfCitingArticles.do?product=WOS&search_mode=NonSelfCitingTCA&qid=21&action=nonselfCA&SID=C6XYSv4Y8ErSIZwNzgd&viewType=summary&betterCount=8036) |
| **2012** | 9,701 | 9,630 | [7,470](https://apps.webofknowledge.com/TotalCitingArticles.do?product=WOS&search_mode=TotalCitingArticles&qid=19&action=totalCA&SID=C6XYSv4Y8ErSIZwNzgd&viewType=summary&betterCount=7470) | [7,430](https://apps.webofknowledge.com/NonSelfCitingArticles.do?product=WOS&search_mode=NonSelfCitingTCA&qid=19&action=nonselfCA&SID=C6XYSv4Y8ErSIZwNzgd&viewType=summary&betterCount=7430) |
| **2013** | 10,284 | 10,176 | [7,881](https://apps.webofknowledge.com/TotalCitingArticles.do?product=WOS&search_mode=TotalCitingArticles&qid=17&action=totalCA&SID=C6XYSv4Y8ErSIZwNzgd&viewType=summary&betterCount=7881) | [7,823](https://apps.webofknowledge.com/NonSelfCitingArticles.do?product=WOS&search_mode=NonSelfCitingTCA&qid=17&action=nonselfCA&SID=C6XYSv4Y8ErSIZwNzgd&viewType=summary&betterCount=7823) |
| **2014** | 6,090 | 6,054 | 5,088 | [5,060](https://apps.webofknowledge.com/NonSelfCitingArticles.do?product=WOS&search_mode=NonSelfCitingTCA&qid=15&action=nonselfCA&SID=C6XYSv4Y8ErSIZwNzgd&viewType=summary&betterCount=5060) |
| **2015** | 7,009 | 6,940 | [5,448](https://apps.webofknowledge.com/TotalCitingArticles.do?product=WOS&search_mode=TotalCitingArticles&qid=13&action=totalCA&SID=C6XYSv4Y8ErSIZwNzgd&viewType=summary&betterCount=5448) | [5,392](https://apps.webofknowledge.com/NonSelfCitingArticles.do?product=WOS&search_mode=NonSelfCitingTCA&qid=13&action=nonselfCA&SID=C6XYSv4Y8ErSIZwNzgd&viewType=summary&betterCount=5392) |
| **2016** | 4,817 | 4,686 | [3,744](https://apps.webofknowledge.com/TotalCitingArticles.do?product=WOS&search_mode=TotalCitingArticles&qid=11&action=totalCA&SID=C6XYSv4Y8ErSIZwNzgd&viewType=summary&betterCount=3744) | [3,668](https://apps.webofknowledge.com/NonSelfCitingArticles.do?product=WOS&search_mode=NonSelfCitingTCA&qid=11&action=nonselfCA&SID=C6XYSv4Y8ErSIZwNzgd&viewType=summary&betterCount=3668) |
| **2017** | 3,327 | 3,239 | 3,239 | 2,379 |
| **2018** | 1,697 | 1,548 | 1,264 | 1,119 |

**Table S2: Citation rate and dynamics**
